# Profile of dermatoses in extreme weather events: case series during floods in the state of Rio Grande do Sul, Brazil^☆^

**DOI:** 10.1016/j.abd.2024.12.001

**Published:** 2025-02-21

**Authors:** Analupe Webber, Cíntia Cristina Pessin, Gabriela Agne Magnus, Guilherme Ladwig Tejada, Isadora da Rosa Hoefel, Jacqueline Sarmento Fernandes, Juliana Catucci Boza, Juliano Peruzzo, Marcelo Balbinot Lucca, Mariele Bevilaqua, Monica Zechmeister Berg, Nathália Hoffmann Guarda Aguzzoli, Renata Alves Sanseverino, Rosemarie Mazzuco, Taciana Dal’Forno Dini, Vanessa Santos Cunha, Veronica Hamann Aita, Renan Rangel Bonamigo

**Affiliations:** a Regional of Rio Grande do Sul, Sociedade Brasileira de Dermatologia, Porto Alegre, RS, Brazil; b Dermatology Service, Santa Casa de Misericórdia de Porto Alegre (ISCMPA), Porto Alegre, RS, Brazil; c Dermatology Service, Hospital de Clínicas de Porto Alegre, Porto Alegre, RS, Brazil; d Postgraduate Program in Medicine, Medical Sciences, Universidade Federal do Rio Grande do Sul, Porto Alegre, RS, Brazil; e Dermatology Service, Pontifícia Universidade Católica do Rio Grande do Sul, Porto Alegre, RS, Brazil

**Keywords:** Climate changes, Dermatology, Extreme weather, Floods, Refugees, Skin diseases

## Abstract

**Background:**

The skin is the first organ of the human body to be exposed to flood water, with local and possibly systemic consequences. There are no Brazilian data on dermatological diseases during recent climate catastrophes related in the country.

**Objectives:**

To assess the demographic profile and dermatological diagnoses in people displaced from their homes and sheltered in collective housing and among rescue workers during the extreme climate crisis in the state of Rio Grande do Sul, Brazil, in 2024.

**Methods:**

This was a cross-sectional and observational study. Information was collected in person or through records, retrospectively.

**Results:**

Data were collected from 371 people with dermatological complaints, and a total of 423 dermatoses were diagnosed. The most prevalent dermatological diseases were dermatoparasitosis, pyoderma, and skin conditions due to trauma and/or injuries. The male gender was statistically associated with traumatic dermatoses/injuries, and females with pyoderma (p < 0.05).

**Conclusion:**

In the recent episode of extreme climate crisis in Brazil, infectious and traumatic dermatoses were the most prevalent among the affected persons. The role of dermatologists in providing care for this population, as well as guiding other colleagues in the management of skin diseases during the floods is highlighted.

**Study limitations:**

The study was conducted in shelters, and some data were evaluated retrospectively. No complementary exams were used for diagnosis.

## Introduction

The state of Rio Grande do Sul (RS) in Brazil experienced the greatest climate crisis in its history in April and May 2024. Rainfall levels were extremely high, and institutional and civil society strategies were insufficient to prevent highly destructive scenarios – a catastrophe that caused extensive damage to the society.

The most serious and direct impact was on the lives of people affected by urban and rural flooding in certain areas of the state. By the end of May 2024, the number of recorded deaths was close to two hundred people, with many missing and more than 600,000 people displaced from their homes.^1^

Among those displaced were the ones rescued and those who moved from their homes and went to shelters – which were organized by an extensive network of private and public institutions – located in the capital city of Porto Alegre, and in other affected cities. The operation and logistics, from rescue to shelter maintenance, were carried out by volunteers and employees of public and private institutions.

Contact with flood water – from rivers, streams, lakes, lagoons, and canals – mixed with organic waste, different types of materials, and sewage, affected many displaced people and rescue workers. Moreover, the various circumstances associated with floods, such as accidents during transportation, incidents during rescues, exposure to cornered animals, landslides and collapsed structures affected many people (displaced persons and rescue workers).

**Fig. 1:**
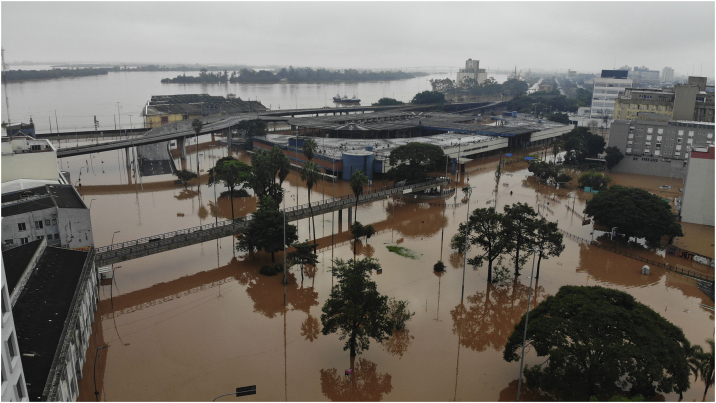
Flooding of Porto Alegre, on the Guaíba Lake ‒ photograph by Douglas Rohers.

**Fig. 2:**
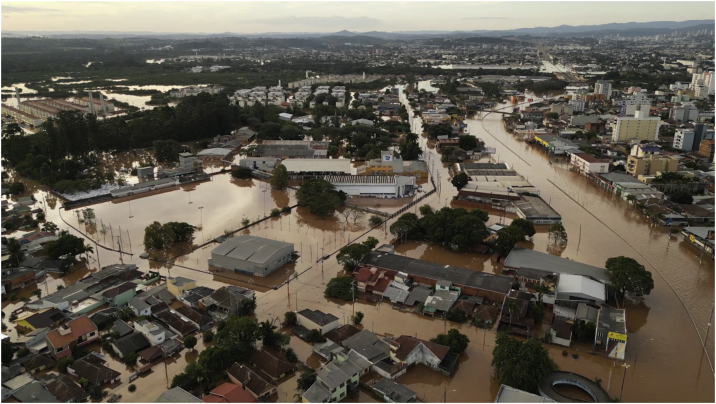
Flooding of São Leopoldo, on the Sinos River – photograph by Douglas Garcia.

**Fig. 3:**
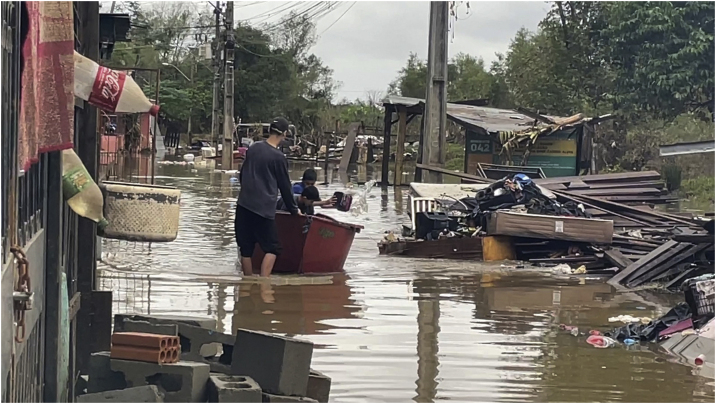
Flooding, sewage and garbage in the city of Novo Hamburgo, on the local stream – photograph by Simone Feltes.

**Fig. 4:**
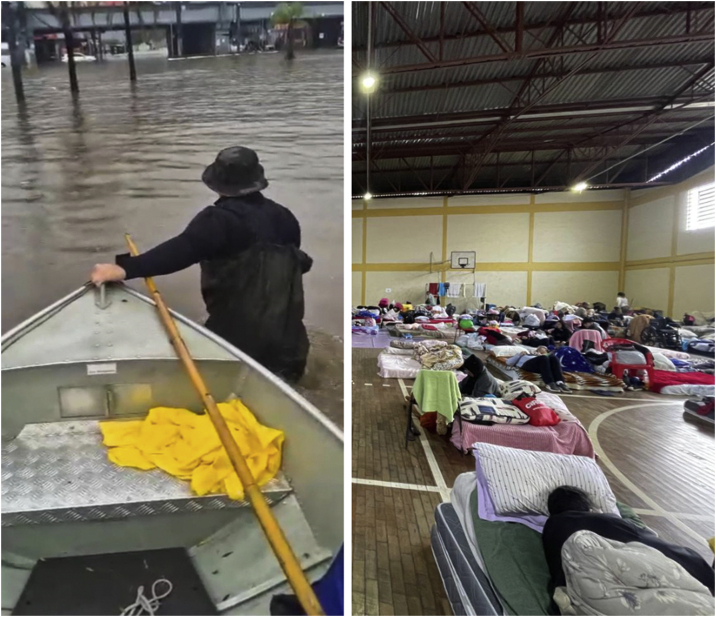
(A) Rescue worker during the floods in Rio Grande do Sul. (B) Inside of a shelter – photographs by Patrick Nascimento and Guilherme Ladwig Tejada, respectively.

**Fig. 5:**
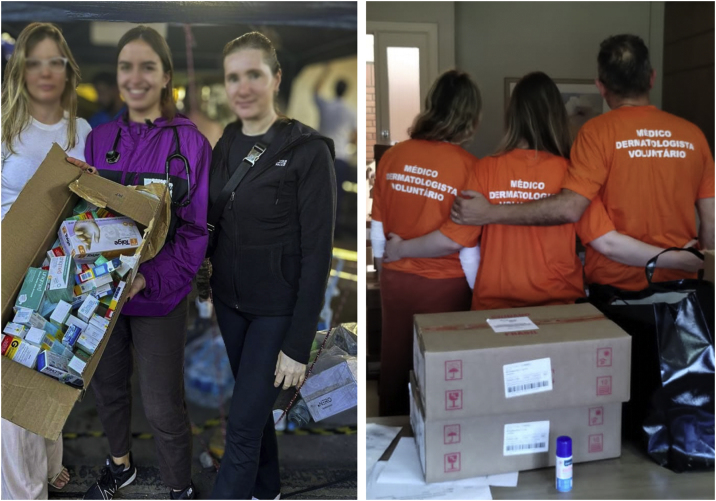
(A) Dermatology volunteers, with donations of medicines in the screening and reception area. (B) Dermatologists from SBD-RS united to help the homeless – photographs by Mariele Bevilaqua and SBD-RS, respectively.

Fig. 1, Fig. 2, Fig. 3 illustrate the immediate consequences of floods, affecting cities, people, flora and animals. Fig. 4, Fig. 5 show rescue workers, the inside of a shelter and volunteer doctors.

The skin is the first organ of the human body to get in contact with flood water and skin health can be compromised, with local and eventually systemic consequences.^2^

This study, developed by volunteer dermatologists from the Brazilian Society of Dermatology – Rio Grande do Sul Sector, aimed to evaluate people affected by floods who developed dermatological diseases, providing important data for the knowledge of the scientific community and the society in general.

## Method

With the aim of evaluating the profile of dermatoses among people displaced from their homes and sheltered in collective housing and among rescue workers, an observational, cross-sectional study of a series of cases was designed. Data were collected prospectively and retrospectively (files/medical records from shelters and/or volunteer doctors) by dermatologists and volunteer resident doctors, using a structured questionnaire. The research was approved by the Research Ethics Committee of the Hospital de Clínicas de Porto Alegre (CAAE 80193524900005327). The Informed Consent Form (FIC) was used for prospective cases and waived for retrospective cases.

The collected variables evaluated comprised age, gender, city where the shelter was located, city where the rescue took place, type of service (in-person or online), participant condition (sheltered: persons displaced by the 2024 climate crisis in Rio Grande do Sul referring to a group of people affected by the floods; rescue worker: person who helped rescue people affected by the 2024 climate crisis in Rio Grande do Sul; sheltered and rescuer: sheltered person who became a rescue worker or rescuer who became a sheltered person) and dermatoses. These were classified as main dermatosis and other dermatoses (diagnosed during physical examination, but not the participant main disease). The dermatoses were grouped as infectious, inflammatory, traumatic/injury-related, and miscellaneous.

Operation and statistical analysis: a questionnaire prepared in Google Forms was completed by dermatologists and resident doctors, volunteers, who worked in shelters in the city of Porto Alegre and cities in the metropolitan region. The variables were entered into Excel and analyzed using a recent version of the SPSS software. The frequencies of dermatoses were evaluated in absolute numbers and percentages. The other variables were described in the univariate analysis. Associations between variables were evaluated using Pearson's chi-square test. The ANOVA method evaluated variances and the post-hoc tests (Tukey's test) were subsequently performed for distinct findings between groups of dermatoses. Significance levels were defined as p < 0.05.

## Results

A total of 371 people were evaluated in different shelters in the city of Porto Alegre and the metropolitan region. During the study period, approximately 14,000 people were sheltered.^1^

Age showed a normal distribution according to the Kolmogorov-Smirnov test, and the mean was 30.96 years, ranging from 0 to 83 years, with a standard deviation of 20.05 years. Among the participants, 189 (50.9%) identified themselves as females and 182 (48.8%) as males. One person (0.3%) preferred not to declare gender.

Three hundred and twenty-seven (88.1%) participants were evaluated retrospectively, through medical records, and 44 (11.9%) participants were prospectively evaluated. Of the total, 350 (94.3%) were sheltered people, 9 (2.4%) were rescue workers, and 12 (3.2%) were both sheltered and rescue workers. Care was provided in person for 362 people (97.6%) and via teleconsultation for 9 (2.4%) people.

As for the city of origin, 234 (63.1%) people were from Porto Alegre, 44 (11.9%) from cities in the metropolitan region, 3 (0.8%) from municipalities in the interior of Rio Grande do Sul, and 89 people (24%) did not have their city of origin identified.

Different types of dermatoses were diagnosed and the main dermatosis in each evaluated person was described. In addition to main dermatosis, 48 ​​people had a second dermatosis diagnosed and three had three dermatoses. The total number of dermatoses in the 371 participants is shown in Table 1.

**Table 1:** Main dermatosis (n = 371) and total dermatoses (n = 423), including the main one and those diagnosed during the physical examination, in the flooding period in Rio Grande do Sul, Brazil, 2024.

Dermatosis	Main dermatosis, n (%)	Total dermatoses, n (%)
Pediculosis	62 (16.7%)	65 (15.4%)
Trauma and injuries (including abrasions, lacerations, cuts caused by trauma)	57 (15.4%)	59 (13.9%)
Bacterial infections (impetigo, cellulitis, erysipelas, donovanosis, syphilis)	47 (12.7%)	60 (14.2%)
Other dermatoses with diagnosis	35 (9.4%)	43 (10.2%)
Superficial and deep mycoses (dermatophytosis, sporotrichosis)	34 (9.2%)	41 (9.7%)
Scabies	23 (6.2%)	26 (6.1%)
Insect bite	17 (4.6%)	20 (4.7%)
Dermatoviruses (herpes simplex and herpes zoster)	16 (4.3%)	17 (4%)
Contact dermatitis (including abrasions caused by pruritus due to flood water)	16 (4.3%)	20 (4.7%)
Unspecified dermatoses	16 (4.3%)	16 (3.9%)
Seborrheic dermatitis	11 (3%)	15 (3.5%)
Dog and cat bites	9 (2.4%)	9 (2.1%)
Pruritus without identifiable cause	8 (2.2%)	8 (1.9%)
Atopic dermatitis	5 (4%)	17 (4%)
Diaper rash	3 (0.8%)	4 (0.9%)
Onychocryptosis	2 (0.5%)	3 (0.7%)

Dermatoses were grouped into infectious (n = 182 or 49.2%), inflammatory (n = 45 or 12.2%), traumatic/injuries (n = 83 or 22.4%) and miscellaneous/other dermatoses, varied or unspecified (n = 60 or 16.2%). Among those in the last group are “skin lesions” (unspecified), keloids, lupus, pemphigus, mouth ulcers, onychopathy, granulomas, skin cancer, burns, and vasculitis.

Table 2 shows the distribution of these groups according to gender (n = 370).

**Table 2:** Main Dermatosis Groups according to gender in sheltered individuals and rescue workers during the flooding period in Rio Grande do Sul, Brazil, 2024 (n = 370).

Dermatoses	Females	Males	Total
Infectious^a^	107 (56.6%)	75 (41.4%)	182 (49.2%)
Inflammatory	26 (13.8%)	19 (10.5%)	45 (12.2%)
Trauma and wounds^a^	31 (16.4%)	52 (28.7%)	83 (22.4%)
Miscellaneous	25 (13.2%)	35 (19.3%)	60 (16.2%)
Total	189	181	370

a p < 0.05.

**Fig. 6:**
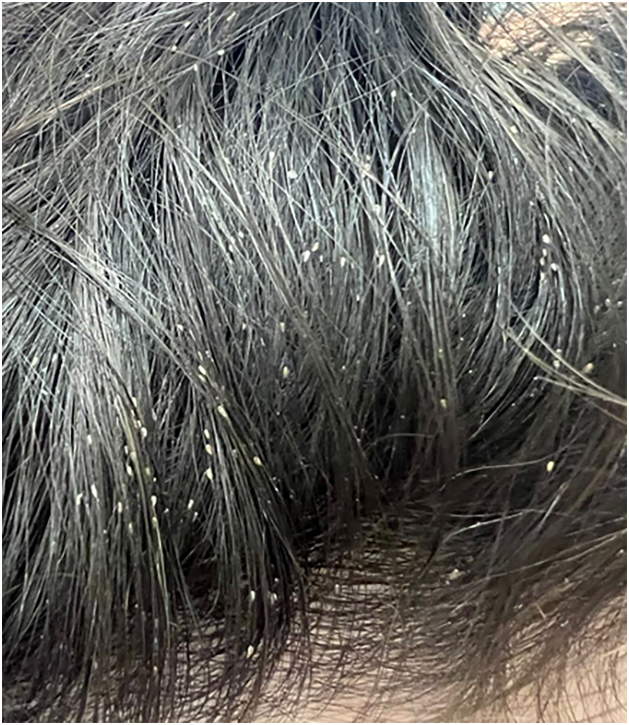
Pediculosis (nits), the most often diagnosed condition in shelters.

**Fig. 7:**
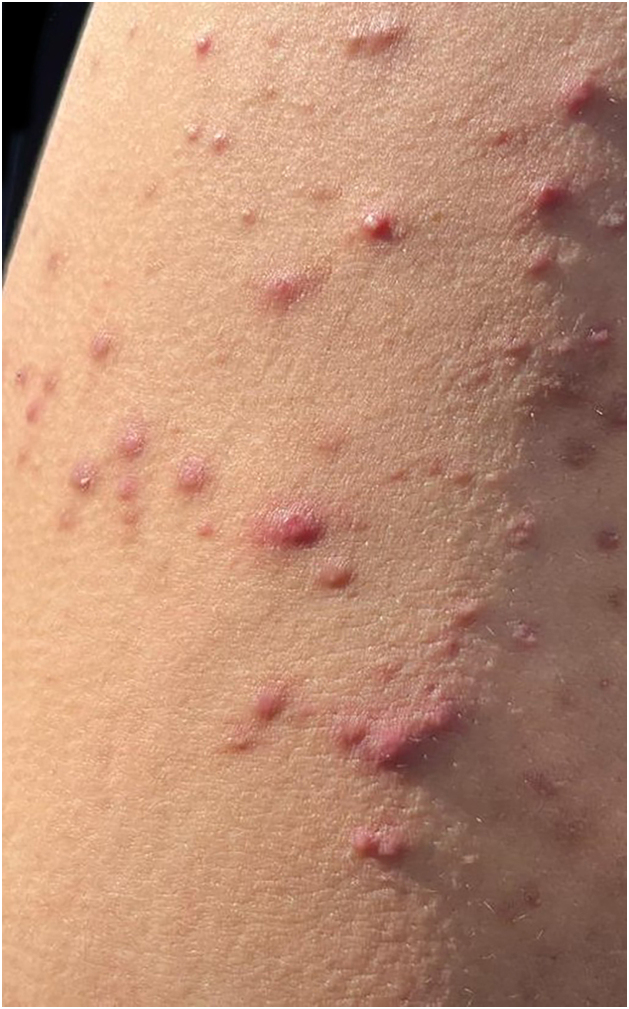
Folliculitis in a rescue worker.

**Fig. 8:**
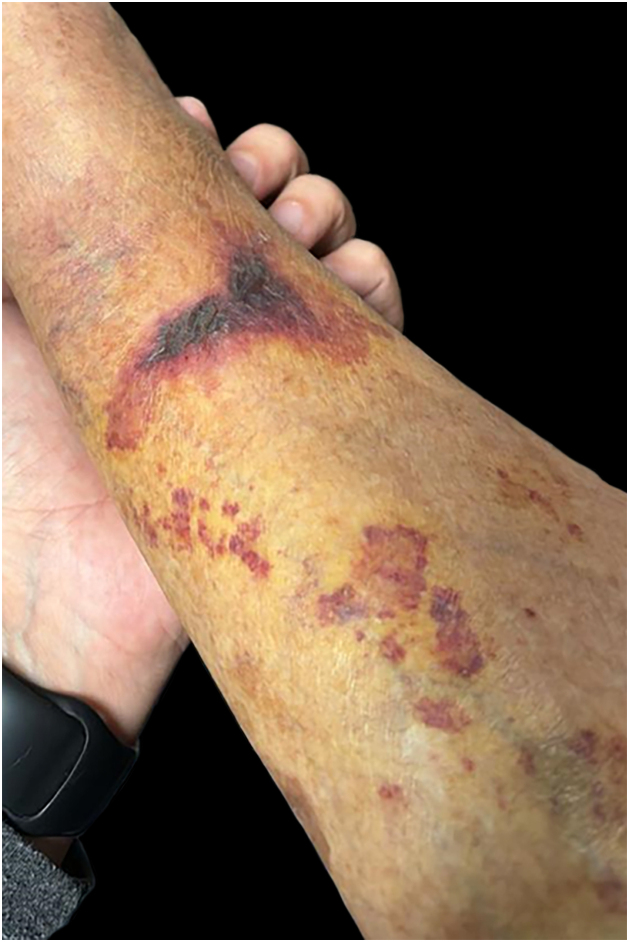
Bruises from trauma, after rescue.

**Fig. 9:**
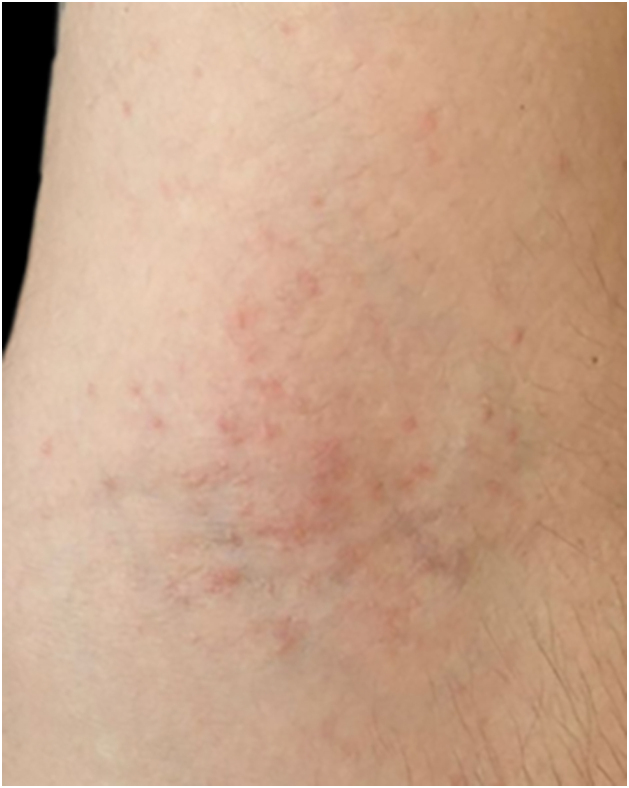
Irritant dermatitis after contact with flood water.

Fig. 6, Fig. 7, Fig. 8, Fig. 9 show examples of the main conditions found, respectively: pediculosis, bacterial infection, injuries after trauma during rescues, and irritant dermatitis due to contact with water and debris.

Using Pearson's Chi-Square test, it was found that infectious dermatoses prevailed in females and the trauma/injury group in males (p < 0.05). The ANOVA analysis of variance showed that there were differences between the ages that affected the different groups of dermatoses (p < 0.05), and in the post-hoc tests, using the Tukey test, it was found that participants in the miscellaneous or unspecified group were older than in the three other groups, with p < 0.05. The mean ages among patients with infectious, inflammatory, and traumatic dermatoses were 29.4 years, 24.0 years, and 31.6 years, respectively, while those with miscellaneous or unspecified dermatoses had a mean age of 40.3 years.

## Discussion

Floods are water overflows caused by hydrometeorological and geophysical disasters, and are the most frequent type of climate disaster (accounting for 40% of calamities) at the global level, with more than 50,000 deaths recorded in the last decade.^3^

In addition to the direct humanitarian disaster, with loss of life and the appearance of climate refugees, the consequences of floods include urban and rural destruction and economic damage hampering the restructuring of the multiple pillars of the affected societies.

The scenario of an increase in the occurrence of extreme events is set, with rising temperatures, rising sea levels, and greater rainfall ‒ in intensity and frequency ‒ as warned by science for decades, and it is up to all social forces in countries and global governance institutions to lead a process aimed to achieve a slowdown in these events, an urban reconfiguration and a social and humanitarian protection network for those potentially and effectively affected.

In addition to the major global climate events of the 21st century ‒ such as those that occurred in Bangladesh (2004), the United States of America (2005), Haiti (2010), Japan (2011), Thailand (2011), and Pakistan (2010)^3^, ^4^, ^5^ ‒ what occurred in Brazil, during the last days of April and in the month of May 2024, was recognized as the greatest climate disaster in the country. The above-average rainfall in the state of Rio Grande do Sul within a very short period of time raised the levels of important rivers and these caused serious flooding and inundation. Among the cities affected were the state capital, Porto Alegre, the cities of the metropolitan region, those in the Taquari River Valley, those bordering the Jacuí River, and those in the southern region of the state and on the coast close to Lagoa dos Patos, such as Rio Grande and Pelotas.^1^

More than two million people were affected in some way, in almost all of the cities in the state of Rio Grande do Sul; more than 600,000 people were displaced, there were more than a hundred deaths, and a large number of missing persons were recorded. Fauna and flora, road structures, residential and commercial buildings, educational and health systems, and different areas of the economy were severely affected by the extreme climate crisis in RS.^1^

In the context of floods, a series of pathogenic factors present as potentially harmful for human health. There is potential contamination of drinking water and food reservoirs, contact with waste and chemical materials, and exposure to microbial agents mainly through the respiratory and digestive systems, and through contact and breakdown of the skin-mucosal barrier. Additionally, psychological implications are very present and should be among the health conditions to be monitored.

Among the most frequently found diseases are those affecting the skin, mucous membranes, and skin appendages. In a study carried out in Pakistan, 28% of those affected by flooding had skin conditions.^7^ The period between the fourth and 28th day after the disaster is the period of greatest risk for wound and trauma infection and the spread of infectious diseases. In addition to skin infections, changes caused by immersion, contact dermatitis, and exacerbation of pre-existing skin conditions are well-documented manifestations after flood disasters.^8^

Another study with firefighters who rescued the victims of the Katrina hurricane in the United States in 2005 and had contact with flood water showed that skin rashes were the most often reported symptoms among those affected, followed by respiratory and gastrointestinal symptoms.^9^ Moreover, it was observed that symptoms were more frequent in those who had prolonged contact with the water and among those who had contact with the water through the nose/mouth or eyes.^9^

The fact that the skin is immediately exposed to a series of products from contaminated water, added to the difficulty of maintaining hygiene in flood conditions, facilitates the onset of skin diseases, which can be grouped into the categories of inflammatory, infectious, traumatic and miscellaneous diseases.

In the current literature on the subject, the most common dermatoses in each group are:^6^, ^7^, ^10^, ^11^, ^12^

- Inflammatory: Contact dermatitis, pruritus, miliaria, pruritus;
- Infectious/Infestations: Bacterial/pyoderma (impetigo, cellulitis, folliculitis, furunculosis, carbuncle, abscesses), fungal (dermatophytosis, candidiasis, deep mycoses), parasitic diseases (scabies, pediculosis, cutaneous hookworm, cutaneous leishmaniasis, amoebiasis, strongyloidiasis, filariasis, onchocerciasis, trypanosomiasis, among others), cercarial dermatitis. The etiologies vary according to local geography and infectious epidemiology;^6^, ^7^, ^10^, ^11^, ^12^
- Traumatic: Lacerations, cut-bruise injuries (bleeding and secondary infections as consequences);
- Miscellaneous: Reactions to mosquito, ant, and arthropod bites (which can cause local and systemic reactions), dog bite injuries (which can be infected by canine flora and/or transmit serious diseases, such as rabies), snakebites and their consequences, immersion foot syndrome, and psychodermatoses.
  In addition to these groups of dermatological diseases, there are pre-existing conditions that are aggravated by the climate disaster (such as atopic dermatitis, psoriasis, urticaria/angioedema, connective tissue diseases, autoimmune bullous diseases) and those that can be induced later, due to the influence of post-disaster psychological conditions, such as vitiligo, alopecia areata, psoriasis and urticaria.^8^, ^9^
  Two dermatological conditions of greater clinical-epidemiological relevance following the floods should be highlighted:
- Inflammatory: Contact dermatitis is the most common inflammatory syndrome. The skin barrier, affected by prolonged immersion and contact with products that cause epithelial damage, allows greater permeability, and various substances come into contact with the inner skin. An acute, immediate inflammatory response is induced by the keratinocyte release of inflammatory mediators. Depending on the duration and composition of the water, the acute phase lasts for days with symptoms such as burning (more than pruritus), erythematous, vesicular, and eroded lesions. The hands and feet are the most affected areas. The possibility of superimposing infections occurs.^12^
- Skin infections/infestations: The introduction of microbial agents via the cutaneous-mucosal integument is a major concern during and after floods. Bacterial infections are most often polymicrobial. The main causes of mild or severe pyoderma are *Staphylococcus sp*, *Corynebacterium sp*, *Aeromonas sp*, *Escherichia coli*, *Klebsiella pneumoniae*, *Pseudomonas aeruginosa*, *Proteus*, *Clostridium*, Burkholderia pseudomallei, Vibrio sp (flooding by seawater), *Leptospira*, *Streptococcus sp*, *Chromobacterium violaceum*, *Mycobacterium ulcerans* and other non-tuberculous mycobacteria. Clinically, there may be lesions of impetigo, cellulitis/erysipelas, abscesses, nodules, gummas and ulcerated tumors.^10^, ^12^, ^13^

Among fungal infections, the most prominent are those caused by dermatophytes, non-dermatophytes, yeasts, and agents of deep mycoses, such as *Fonsecaea pedrosoi*, *Blastomyces spp*, *Mucor spp*, *Rhizopus spp*, *Absidia spp*. Clinically, tinea pedis/interdigitalis/manuum/inguinum are the most common forms of presentation, as dermatophyte infections predominate.^12^, ^13^

The overlap of agents is very common, including bacterial and fungal infections in the same site.^11^, ^12^, ^13^

Among the infestations, scabies and pediculosis stand out, both of which have their spread facilitated by the large human population in collective housing after a disaster.^10^

Scabies can cause intense pruritus and become a risk for serious bacterial infections, particularly in people who were previously immunosuppressed due to nutritional conditions, diseases, or medications. Pediculosis is cited as the most frequent infestation, affecting adults, and particularly children. As excoriations are common, it also becomes a risk for overlapping infections.^10^

After identifying dermatological diseases, it is important that therapeutic management be rapidly implemented to prevent their spread and worsening.^13^

The present study describes dermatological findings in a series of cases during the largest climate crisis in a large Brazilian region. The age profile of those sheltered and/or rescue workers was identified (mean and median age around 30 years, with a wide age range), both males and females being equally affected. A total of 371 people with dermatoses were evaluated. Among the main dermatoses present or identified on physical examination, a total of 423 dermatological diagnoses were made by the medical team. Dermatologists evaluated the patients prospectively or retrospectively, using forms/medical records, which were filled out by doctors, whether dermatologists or not. Other limitations of the study include the fact that the diagnosis was always clinical, as there was no possibility of using complementary exams and, as it was not a cohort study (longitudinal), there was no follow-up of the patients in the medium to long term (other dermatoses could have appeared within weeks or months).

Among the dermatoses identified, pediculosis, bacterial infections and/or traumatic infections/injuries stand out. After classification as inflammatory, infectious, traumatic, and miscellaneous, it was observed that infectious diseases were the most frequent, corresponding to almost half of the total.

Pediculosis – the most frequent dermatosis diagnosed in this study – certainly stood out due to the characteristics of the shelters, where very close grouping of people was extremely common. Besides scabies, it was found that more than 20% of the people were affected by some dermatoparasitosis.

Trauma and injuries resulting from floods and rescue operations were the second most prevalent condition as the main dermatosis, followed by bacterial infections. Additionally, a wide range of other dermatological diseases, many inflammatory and miscellaneous, including pre-existing ones, were observed.

Inflammatory dermatoses – much highlighted in the literature – appeared, but without much prominence. There was probably underdiagnosis in this group since the study did not cover the entire range of affected individuals and diagnoses were made based on people that sought medical assistance.

The impossibility of using complementary diagnostic resources and/or evaluation by non-specialist doctors probably contributed to the fact that a number of dermatoses included in the miscellaneous group were not diagnosed with certainty, and were listed as “unspecified dermatoses” (n = 16 or 3.9% of the total).

Regarding the observed associations, infectious dermatoses were more frequent in females and the trauma/injury group in males (p < 0.05). There is no way to be certain of the reasons for these differences, but it can be assumed that men were more intensely involved in rescue operations and that women were in contact with contaminated water for longer periods.

For decades, science has indicated that society and governments should organize actions that have an impact on the planet climate and its viability. According to Menegat R, Porto ML, Carraro CC and Fernandes LAD, there are necessary measures to empower governance in promoting global sustainability, including: a) Implementing public sector policies that promote sustainable resource management and social development, and b) Committing the private sector, through agreements and programs, to respect and support local strategies for sustainable development in areas in which they invest and operate.^14^

Children and the elderly, as well as individuals with comorbidities or immunosuppression, are at greater risk of suffering from flood-related illnesses.^8^ It has also been shown that the lower-income population is more vulnerable to the effects of disasters, as are the black and Hispanic populations, usually because they live in areas that are more susceptible to the effects of water.^8^ In fact, public policies to improve the general health of these populations and, especially, their housing conditions, including viable housing alternatives that reduce the occupation of high-risk areas, are of fundamental importance.

This study has unique characteristics and historical singularity: it was developed in the midst of an extreme climate crisis, reflects a large movement of volunteer dermatologists and the Brazilian Society of Dermatology, Rio Grande do Sul sector, and despite its intrinsic limitations, it can become a reference for future actions that may be necessary. As limitations, it is known that the research was carried out in shelters, with part of the data evaluated retrospectively. Moreover, complementary exams were not used in the diagnoses and it also did not include patients who required hospitalization.

## Conclusion

In the largest episode of extreme climate crisis in Brazil, the floods and inundations in the state of Rio Grande do Sul in 2024, Dermatology was a very important specialty in the care of those affected, as various skin diseases occurred, mainly infectious ones (particularly pediculosis, bacterial diseases, mycoses and scabies), traumatic/wound-related and inflammatory diseases. Infectious skin diseases were more prevalent in women, while traumatic skin diseases were more prevalent in men.

The authors reinforce the role of dermatologists in this context, providing care to those affected, as well as guiding other colleagues in the management of skin diseases occurring during the floods.

## Authors' contributions

Analupe Webber: Actively contributed to data collection and approved the final version of the manuscript.

Cíntia Cristina Pessin: Actively contributed to data collection and approved the final version of the manuscript.

Gabriela Agne Magnus: Actively contributed to data collection and approved the final version of the manuscript.

Guilherme Ladwig Tejada: Actively contributed to data collection and approved the final version of the manuscript.

Isadora da Rosa Hoefel: Actively contributed to data collection and approved the final version of the manuscript.

Jacqueline Sarmento Fernandes: Actively contributed to data collection and approved the final version of the manuscript.

Juliana Catucci Boza: Actively contributed to data collection and approved the final version of the manuscript.

Juliano Peruzzo: Actively contributed to data collection and approved the final version of the manuscript.

Marcelo Balbinot Lucca: Actively contributed to data collection and approved the final version of the manuscript.

Mariele Bevilaqua: Actively contributed to data collection and approved the final version of the manuscript.

Monica Zechmeister Berg: Actively contributed to data collection and approved the final version of the manuscript.

Nathália Hoffmann Guarda Aguzzoli: Actively contributed to data collection and approved the final version of the manuscript.

Renata Alves Sanseverino: Actively contributed to data collection and approved the final version of the manuscript.

Rosemarie Mazzuco: Actively contributed to data collection and approved the final version of the manuscript.

Taciana Dal’Forno Dini: Actively contributed to data collection and approved the final version of the manuscript.

Vanessa Santos Cunha: Actively contributed to data collection and approved the final version of the manuscript.

Veronica Hamann Aita: Actively contributed to data collection and approved the final version of the manuscript.

Renan Rangel Bonamigo: Actively contributed to data collection and approved the final version of the manuscript.

## Financial support

The study was supported by Sociedade Brasileira de Dermatologia - Regional Rio Grande do Sul (SBDRS).

## Conflicts of interest

None declared.
